# Ameliorative effect of *Lactobacillus rhamnosus* GG on acetaminophen-induced hepatotoxicity via PKC/Nrf2/PGC-1α pathway

**DOI:** 10.1186/s43141-022-00422-4

**Published:** 2022-10-06

**Authors:** Hend M. Ahmed, Hanan H. Shehata, Gamila S. M. El-Saeed, Hoda H. Abou Gabal, Sherien M. El-Daly

**Affiliations:** 1 Medical Biochemistry Department, Medicine and Clinical Studies Research Institute, National Research Centre, Dokki, Cairo, 12622 Egypt; 2 Medical Biochemistry and Molecular Biology Department, Faculty of Medicine, Ain Shams University, Cairo, Egypt; 3 Pathology Department, Faculty of Medicine, Ain Shams University, Cairo, Egypt; 4 Cancer Biology and Genetics Laboratory, Centre of Excellence for Advanced Sciences, National Research Centre, Cairo, Egypt

**Keywords:** Hepatotoxicity, Acetaminophen, Probiotic, *Protein Kinase C*, Oxidative stress, Nrf2, PGC-1

## Abstract

**Background:**

Acetaminophen (APAP) overdose is a common cause of hepatotoxicity. Antioxidants like N-acetyl cysteine are recommended as a therapeutic option; nevertheless, it has limitations. The search for efficient alternatives is ongoing. Probiotics are live microorganisms that maintain a healthy gut microecology. *Lactobacillus rhamnosus* GG (LGG) is one of the widely used probiotics. Our study aimed to assess the protective and therapeutic effects of probiotic LGG on APAP-induced hepatotoxicity and evaluate the molecular pathways behind this effect.

**Methods:**

Wistar Albino male rats were randomly distributed into the following experimental groups: group 1, non-treated rats (vehicle); group 2, rats received oral gavage of suspension of probiotic LGG (5 × 10^10^ CFU GG/0.5 ml in PBS) daily for 2 weeks (probiotic control); group 3, rats received APAP dose of 2 g/kg body weight (positive control); group 4, rats received oral gavage of suspension of probiotic LGG for 2 weeks followed by a single dose of APAP injection (prophylactic); and group 5, rats received a single dose of APAP and then 24 h later treated with oral gavage of probiotic LGG daily for 2 weeks (treatment).

**Results:**

Our study revealed that administration of probiotic LGG (either as prophylactic or treatment) exhibited a remarkable reduction in APAP-induced liver injury as resembled by the decrease in liver enzymes (ALT and AST) and the histopathological features of liver sections. Moreover, the significant reduction in the oxidative marker malondialdehyde, along with the enhancement in glutathione reductase, and the significant reduction in inflammatory markers (nitric oxide and tumor necrosis factor-α) were all indicators of the efficiency of LGG in ameliorating the alterations accompanied with APAP-induced hepatotoxicity. Our findings also demonstrate that LGG administration boosted the expression of Nrf2 and PGC-1 while decreasing the expression of protein kinase C (PKC). As a result, the nuclear abundance of Nrf2 is increased, and the expression of various antioxidants is eventually upregulated.

**Conclusion:**

Our study shows that probiotic LGG supplementation exerts a prophylactic and therapeutic effect against APAP-induced hepatotoxicity through modulating the expression of PKC and the Nrf2/PGC-1α signaling pathway and eventually suppressing oxidative damage from APAP overdose.

## Background

Drug-induced liver injury represents a serious adverse event associated with several drugs uptake. Acetaminophen (N-acetyl-p-aminophenol, APAP, commonly named Paracetamol) is a well-known antipyretic and analgesic drug. Although it is effective and safe at therapeutic doses, an overdose may lead to hepatotoxicity and acute liver damage [1, 2]. Paracetamol intoxication is reported to be responsible for a high number of acute liver failure cases and thus has grown to be a significant public health concern. APAP poisoning is responsible for 46% of acute hepatotoxicity in the USA and 40–70% of all cases in the UK and Europe over the last 40 years [3]. According to the primary poison control center at the Ain Shams University Hospitals in Egypt, Paracetamol is one of the top ten most commonly involved exposure compounds [4].

At therapeutic doses, acetaminophen is metabolized hepatically by sulfation and glucuronidation and converted to nontoxic metabolites. The cytochrome P450 enzymes oxidize a minor amount of acetaminophen to the reactive metabolite N-acetyl-p-benzoquinone-imine (NAPQI) in the liver. In cases of APAP overdose, the phase II biotransformation pathway is decreased, and the activity of cytochromes is increased, generating higher amounts of NAPQI in hepatocytes, leading to ACUTE liver damage.

In the case of acetaminophen toxicity, high amounts of NAPQI are produced, depleting glutathione storage in the liver, thus causing oxidative stress, mitochondrial dysfunction, and decreased adenosine triphosphate, leading to liver toxicity [5–7]. Previous studies have explored the role of antioxidants in protecting the liver from APAP intoxication. It was reported that N-acetylcysteine, and activated charcoal, in addition to some natural products and herbs, has protective and therapeutic roles against APAP-induced hepatotoxicity [8, 9].

Live microbial food supplements, known as probiotics, have been reported to provide health benefits to the host when consumed in appropriate proportions. Probiotics are safe, well-accepted by the public, and have recently been considered promising natural treatments. Probiotics are generally considered safe for human administration by the US Food and Drug Administration (FDA) and have received a qualified presumption of safety (QPS) status by the European Food Safety Authority (EFSA) [10]. Probiotics are accessible in the form of functional meals, supplements, and pharmaceuticals. It is available in a wide range of commercial products with diverse doses and microbial components (tablets, capsules, vials, supplements, milk formulas, etc.). Probiotics’ therapeutic benefits on a number of health conditions have been extensively researched in different animal models and clinical investigations. Studies have demonstrated the efficacy of probiotics against several diseases, such as gastrointestinal bacterial infection [11], hepatic encephalopathy [12], alcohol-induced liver toxicity [13], and nonsteroidal anti-inflammatory drug enteropathy [14], as well as promising anti-tumor activity [15, 16]. These beneficial effects result from restoring the balance between commensal and pathogenic gastrointestinal flora, inhibiting harmful bacteria by altering the intestinal environment, reducing ammonia and endotoxin levels in the liver, increasing vitamin synthesis, and reducing cholesterol levels. Moreover, probiotics inhibit the growth of harmful bacteria by producing free fatty acids and antimicrobial peptides [17–19].

Probiotics primarily consist of lactic acid bacteria, such as *Lactobacillus*, *Enterococcus*, *Streptococcus*, and *Bifidobacterium*. The *Lactobacillus rhamnosus* GG (LGG), a gram-positive bacterium, is well used probiotic strain because of its numerous beneficial impact on the gastrointestinal barrier and inflammatory profile [20, 21]. Therapeutic effects of LGG have been demonstrated in multiple diseases, including diarrhea, allergy, and fatty liver in preclinical studies [22, 23]. A randomized clinical trial showed that LGG was safe in patients with liver cirrhosis, reduced endotoxemia, and improved gut dysbiosis in cirrhotic patients [24].

The current study aimed to assess the protective and therapeutic effects of probiotic *Lactobacillus rhamnosus* LGG on acetaminophen-induced hepatotoxicity. We evaluated the modulatory impact of LGG administration on the key molecular markers and pathways involved in hepatotoxicity from oxidative stress to inflammation. Our study revealed the efficiency of probiotic LGG to improve most signs of acetaminophen-induced hepatotoxicity. Interestingly administration of probiotic LGG was also able to prevent the expected damaging effect of acetaminophen, indicating a prophylactic efficiency of probiotic LGG.

## Methods

### Animals and materials

Wistar Albino male rats (160–180 g body weight) were provided from the Animal Laboratory Unit at the National Research Centre, Giza, Egypt. The animal study protocol was reviewed and approved by the ethical committee, National Research Center (Ethical approval code 19–017). Animal study protocols were in compliance with the guidelines of the NRC animal research ethics committee. Rats were kept in merit conditions, including a 12-h light/dark cycle at 22 °C ± 2 °C, open access to water, and a pellet diet. The animals were acclimated to these terms for 2 weeks before the start of this study.

The probiotic dietary supplement *Lactobacillus rhamnosus* was purchased under the commercial name *L. rhamnosus* 200 billion CFUs per gram from custom probiotics, Inc. Glendale, California. This product is a gram-positive lactic acid bacterium that is found in the normal gut microflora of humans. It is generally recognized as safe and has been widely utilized in food goods and health supplements. It contains no gluten, casein, dairy, sugar, soy, flavors, yeast, artificial colors, preservatives, or genetically modified or altered organisms. It can survive harsh stomach acids to reach the intestinal tract where they exert its greatest benefits. The probiotic mixture was given at the dose 5 × 10^10^ CFU GG/0.5 ml phosphate-buffered saline (PBS), prepared by directly suspending lyophilized bacteria in PBS by oral gavage [22]. Paracetamol (acetaminophen) A5000 meets USP (United States Pharmacopeia) testing specifications, 98.0–102.0% powder; was purchased from Sigma Aldrich. Paracetamol (acetaminophen) dose of 2 g/kg b.wt was applied in our study [25].

### Experimental design

Rats were randomly divided to five groups (*n* = 8 per group) as follows:

- Group 1 (vehicle): Rats served as negative control and only received saline.
- Group 2 (probiotic control): Rats received oral gavage of suspension of probiotic *Lactobacillus rhamnosus* LGG (5 × 10^10^ CFU GG/0.5 ml in PBS) daily for 2 weeks [26].
- Group 3 (positive control): Rats received acetaminophen APAP) dose of 2 g/kg body weight once orally [25, 27].
- Group 4 (prophylactic group): Rats received oral gavage of suspension of probiotic *Lactobacillus rhamnosus* LGG (5 × 10^10^ CFU GG/0.5 ml) daily for 2 weeks, and then on day 14, rats received a single dose of acetaminophen orally (2 g/kg b.wt.).
- Group 5 (therapeutic group): Rats received a single dose of acetaminophen orally (2 g/kg b.wt.) then 24 h later treated with oral gavage of suspension probiotic *Lactobacillus rhamnosus* LGG (5 × 10^10^ CFU GG/0.5 ml) daily for 2 weeks.

At the end of the experiment, blood samples were freshly obtained from the retro-orbital venous plexus of the eye using capillary tubes after adding a topical anesthetic solution to the site of puncture. Blood samples were centrifuged at 3000 rpm for 10 min, and the serum was separated and stored at − 20 °C for further analysis. Rats were killed by decapitation, and liver tissue samples were dissected from all rats for histopathological examinations, measurement of different biochemical markers, and gene expression analysis. For biochemical analysis, fresh liver tissue sections were cut into small pieces and homogenized in phosphate buffer saline (PBS, pH = 7.4), then centrifuged at 4000 rpm for 15 min, and stored at − 20 °C for further biochemical analysis.

### Histopathological and immunohistochemical examination

Liver tissue samples were removed, washed with cold and isotonic saline, and excised into small tissue sections. Liver sections were dried using filter paper and preserved in 10% formaldehyde until further used. Preserved liver tissue was then embedded in paraffin blocks. Paraffin-embedded (FFPE) blocks of tissue were cut into 5-µm-thick sections and stained with the hematoxylin–eosin (H&E stain) for histological examination by light microscope. For immunohistochemical examination, liver tissue sections cut from the formalin-fixed paraffin-embedded blocks were mounted onto positively charged adhesive slides. The tissue slides were subject to immunohistochemical staining, including deparaffinization, rehydration, blocking, and staining with an anti-Nrf2 monoclonal antibody (cell signaling, no. 33649) with rat species reactivity. Stained tissue sections were visualized using a streptavidin–horseradish peroxidase technique. Tissue sections were examined, and images were captured by light microscopy (Axio Imager Z2, Carl Zeiss, Jena, Germany) [28].

### Biochemical analysis

Fresh liver tissue sections were cut into small pieces and homogenized in phosphate buffer saline (PBS, PH = 7.4) and then centrifuged at 4000 rpm for 15 min. The supernatant was separated and stored at − 20 °C for further biochemical analysis.

#### Estimation of liver function biomarkers

We evaluated the levels of liver enzymes (ALT and AST) using commercial kits (Teco Diagnostics, Lakeview Ave., USA) following the colorimetric method previously described by Huang et al. [29]. Serum ALT and AST levels were measured using glutamate dehydrogenase by determining the amount of glutamate formed in 20 μl serum incubated for 45 min at 37 °C. The dehydrogenation of glutamate reduces the diazonium salt, which is measured by absorbance at 520 nm [30]. For each parameter measured colorimetrically, the appropriate blank was considered. Data is calculated and presented as U/L.

#### Estimation of kidney function biomarkers

Urea and creatinine were measured in serum to evaluate kidney function. Serum urea in all samples was detected using commercial colorimetric kits. For urea measurement (BioMed Diagnostic, Cat. no. UR 2110, EGY-CHEM, Egypt), the enzyme urease hydrolyzes urea to ammonia (NH3) and carbon dioxide (CO_2_). In the presence of salicylate and nitroferricyanide, the released ammonium reacts with the alkaline solution (sodium hypochlorite) to form a green dye compound that absorbs at 570 nm [31]. Serum creatinine level was measured using a colorimetric kit (BioMed Diagnostic, Cat. no. UR 1250, EGY-CHEM, Egypt) in which creatinine reacts with picrate ions under alkaline conditions to form an orange complex that is measured at 520 nm [32]. The intensity of the color produced is proportional to the concentration of creatinine in the sample. Data is calculated and presented as mg/dl.

#### Evaluation of oxidant and antioxidant activities

Oxidant and antioxidant activities were estimated in liver tissue homogenates. Liver tissue homogenates were prepared by homogenizing liver samples in PBS solution (pH 7.4) containing 0.16 mg/ml heparin to clean the tissues from clots or red blood cells.

#### Malondialdehyde (MDA)

Malondialdehyde (MDA) was measured in tissue homogenates by a colorimetric method using (Biodiagnostic kit, Cat. no. MD-2529, Egypt) according to a method described earlier [33]. At 95 °C, the chromogen thiobarbituric acid (TBA) interacts with the malondialdehyde in the sample under acidic conditions to form a TBA-reactive pink product. The absorbance of the colored product is measured at 534 nm [34]. Data is calculated and presented as nmol/mg protein.

#### Glutathione reductase (GR)

Glutathione reductase levels were determined using a Biodiagnostic kit (Cat. no. GR 2511, Giza, Egypt) based on the enzymatic method described by Pannala et al. [35]. In the presence of NADPH, GR catalyzes the reduction of glutathione (GSSG), which is then oxidized to NADPH^+^. The decrease in absorbance at 340 nm is tracked kinetically over time. Data is calculated and presented as U/L.

#### Evaluation of inflammatory biomarkers

##### ***Nitric oxide***

Nitric oxide (NO) levels were estimated using a Biodiagnostic kit (Cat. no. 2533, Giza, Egypt). The measurement of total nitrite NO levels in liver tissue homogenates was done for a reliable determination of total NO production. We used a commercial kit that relies on the Griess reaction following the methodology described by Guevara et al. [36]. The Griess reaction produces a colored azo dye substance that absorbs visible light at 540 nm, which is proportional to nitrite concentration. Data is calculated and presented as umol/L.

##### ***Tumor necrosis factor-α (TNF-α)***

The sandwich enzyme-linked immunosorbent assay (ELISA) technique was used to measure serum tumor necrosis factor-α (TNF-α) levels. Detection and quantitation were conducted using a commercially available kit specific for rat TNF-α (Glory Science Co., Ltd. Cat no.:30635). TNF-α in serum samples forms a complex with a target-specific capture antibody coated on the microtiter plate. A second (detector) antibody is then applied to this complex, followed by the addition of a substrate solution that interacts with the enzyme-antibody-target complex to create a detectable signal read at 450 nm [37].

### RNA extraction and real-time PCR for gene expression analysis

Total RNA was extracted from all liver tissue sections using PureLink® RNA Mini Kit-Cat. no. 12183018A (Life Technologies, USA) to efficiently isolate high-quality total RNA while removing the majority of genomic DNA. The quality and quantity of extracted total RNA were assessed using NanoDrop Nucleic Acid Quantification (Thermo Fisher, USA). Extracted RNA samples were reverse-transcribed with RevertAid-RT Reverse Transcription Kit (Thermo Fisher Scientific, Cat. no. K1691, USA) under the following conditions: 60 min at 42 °C and the reaction terminated by heating at 70 °C for 5 min. Real-time PCR was conducted using Quantitect SYBR-Green PCR kit, Cat. no. 204243 (Qiagen, USA) on an Agilent RT-PCR machine (Mx3000P). Primers used in our study were purchased from the Qiagen QuantiTect primers collection as follows:

- *Protein kinase C-alpha (PRKC-α)* primer: Rn_Prkca_2_SG QuantiTect Primer Assay
- *Protein kinase C-gamma (PRKC-γ)* primer: Rn_Prkcg_1_SG QuantiTect Primer Assay
- *Nuclear factor erythroid 2-like2 (Nrf2)* primer: Rn_RGD:620360_1_SG QuantiTect Primer A
- *Peroxisome proliferator-activated receptor-gamma coactivator1 alpha (PGC-1α)* primer: Rn_Ppargc1a_1_SG QuantiTect Primer Assay
- *β-actin* primer: Rn_Actb_1_SG QuantiTect Primer Assay

SYBR Green PCR master mix for one sample was prepared in a total volume of 25 μl by mixing the following: 12.5 μl 2 × QuantiTect SYBR Green PCR Mix + 2.5 μl of QuantiTect Primer Assay (0.5 uM) + 2.5 μl Template cDNA (5–10 ng) + RNase-free water (variable). The cycling conditions used for real-time PCR were set as follows: 15 min at 95 °C, followed by 35 cycles of 94 °C for 15 s, 60 °C for 30 s, and 72 °C for 30 s [38]. Melting curve analysis was performed following each run to assess the dissociation characteristics. Cycle threshold (Ct) values were obtained following amplification, and gene expression for the samples was normalized to the internal control β-actin. The relative mRNA expression was calculated using the comparative 2^−ΔΔCt^ method, and data are represented as relative expression.

### Statistical analysis

The results of the present study are presented as mean ± SD. Statistical analysis was assessed using the statistical software SPSS V16.0. One-way ANOVA followed by Fisher’s least significant difference (LSD) test was performed for statistical significance between different groups. Differences with *p*-values < 0.05 were considered statistically significant.

## Results

### Histopathological examination

**Fig. 1:**
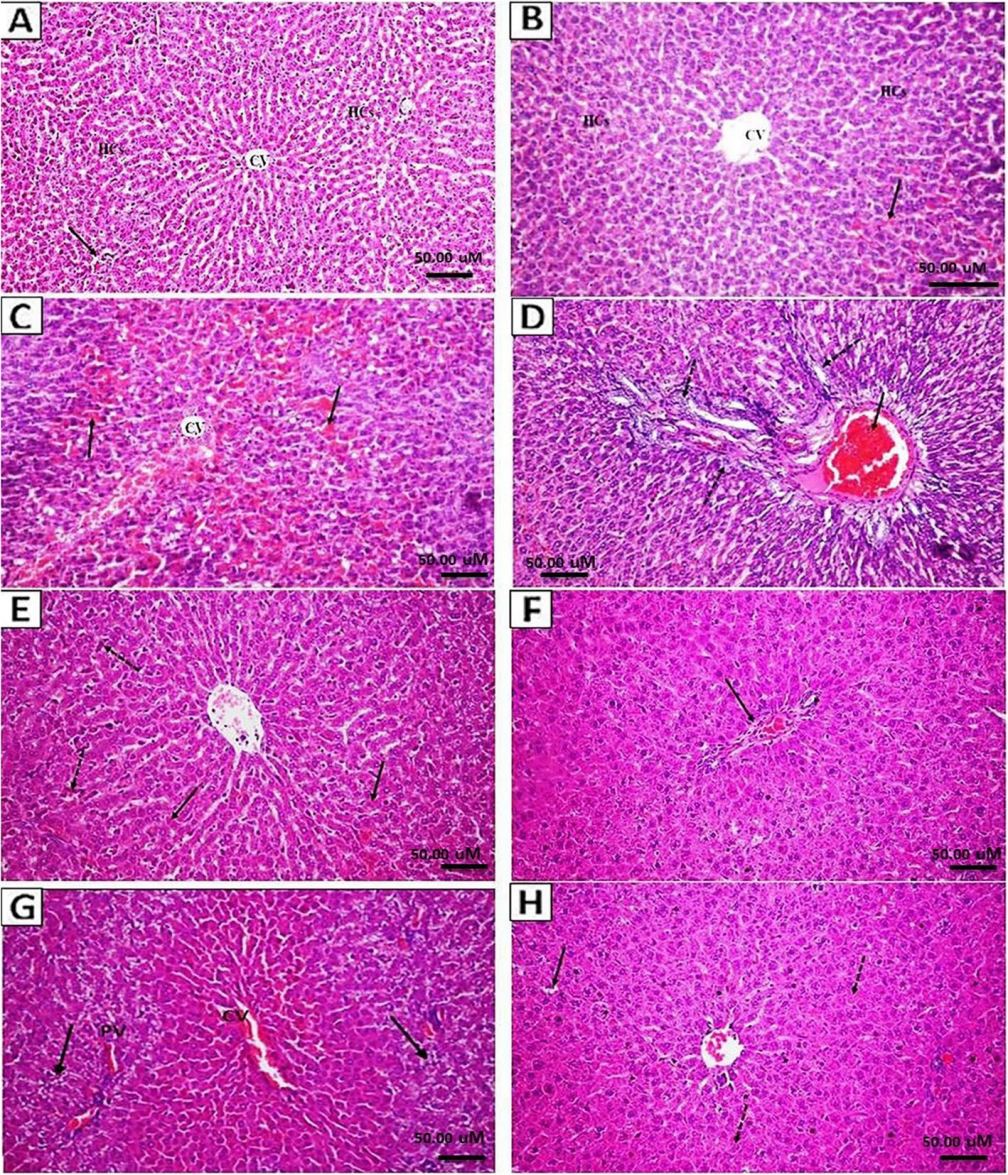
Histopathological features of liver tissue sections from the different study groups. **A**) Liver section from vehicle group shows normal histological structure of central vein (CV), normal hepatic parenchymal cells (HCs), and normal portal area (arrow). **B**) Liver section of probiotic control reveals normal histological structure except congestion of some sinusoids (arrow). **C**) and **D**) Liver tissue sections from APAP-positive control group show severe congestion of the central vein (CV), hepatic sinusoids (arrow), portal vessels congestion, multiple newly formed bile ductules (dashed arrows), and mild fibrosis (asterisk) were also detected. **E**) and **F**) Liver sections from APAP-administrated group received probiotic as a prophylactic dose demonstrates mild degree of hepatocellular degenerative and necrotic changes (arrow), with some leukocytic exocytosis of the hepatic sinusoids (dashed arrow). **G**) and **H**) Liver sections of APAP-administrated rat that received probiotic LGG as treatment show mild congestion of the central vein, hepatic sinusoids, and portal vessels (PV), along with moderate degree of periportal hepatocellular degeneration (arrow). **H**) Hepatocellular swelling, mild scattered vacuolar degeneration (arrow), and necrosis (dashed arrow). H&E staining, magnification 200 ×

Microscopic examination of various liver tissue sections from control rats (vehicle group) revealed a normal histological structure of the central vein, normal hepatic parenchymal cells, and normal portal area (Fig. 1A). Simultaneously, liver sections from normal rat-administrated LGG probiotic (probiotic control group) exhibited a normal histological structure except for congestion of some sinusoids (Fig. 1B). In contrast, liver sections of APAP-administrated rats (positive control group) revealed severe congestion of the central veins and hepatic sinusoids (Fig. 1C). Pericentral hepatocellular necrosis and peripheral vacuolar degeneration were also observed with some cyst formation as well as necrosis. The portal areas displayed congestion of the portal vessel, newly formed bile ductules, and mild fibrosis (Fig. 1D). In the liver of APAP-treated rats which received probiotic LGG as a prophylactic intervention, the examination of which showed good protection of the hepatic parenchyma with a mild degree of hepatocellular degenerative and necrotic changes with some leukocytic exocytosis of the hepatic sinusoids (Fig. 1E). The portal areas in the liver showed only congestion of the portal vessel with near to normal appearance of the portal tract (Fig. 1F). However, the liver of APAP-treated rats which received a therapeutic dose of LGG probiotic (therapeutic group) showed mild congestion of the central vein, hepatic sinusoids, and portal vessels along with mild to moderate degree of hepatocellular degeneration, particularly vacuolar degeneration, and scattered necrosis (Fig. 1 G and H). Semiquantitative histopathologic scoring in the five studied groups is depicted in Table 1.

**Table 1:** Semiquantitative histopathologic scoring in the different studied groups. The H&E stained liver tissue sections were graded using the following scale: ( −) normal, (0/ +) normal or mild, ( +) mild, (+ / + +) mild or moderate (+ +) moderate, and (+ + +) severe

** Groups**	**Blood vessels and sinusoids congestion**	**Inflammatory cellular infiltrate**	**Vacuolar degeneration**	**Parenchymal necrosis**	**Ductular proliferation**	**Fibrosis**
**Vehicle**	−	−	−	−	−	−
**Probiotic control**	+	+	−	−	−	−
**Positive control**	+ + +	+ + +	+ + +	+ + +	+ / + +	+
**Prophylactic**	+	+	+	+	−	−
**Therapeutic**	+ / + +	+ +	+ / + +	+ / + +	− / +	−

### Alterations of biochemical markers following administration of paracetamol and/or probiotic

#### Liver and kidney function biomarkers

Our results revealed that all rats that received paracetamol and were divided into positive control, prophylactic, or therapeutic groups showed significant elevation of ALT, AST, creatinine, and urea compared with the vehicle group (Table 2). The administration of the probiotic LGG as a prophylactic or therapeutic significantly (*p* < 0.05) decreased serum levels of those biomarkers compared with the APAP-positive control group (Table 2). Interestingly, a significant reduction in liver and kidney parameters was most evident in the group that received probiotic LGG as a prophylactic.

**Table 2:** Levels of serum ALT, AST, creatinine, and urea in the different studied groups administered APAP and/or probiotic LGG

** Parameters**	**Vehicle**	**Probiotic control**	**Positive control**	**Prophylactic**	**Therapeutic**
**Serum ALT (U/L)**	**44.75 ± 5.3**	**47.38 ± 7.61**^b^	**82.28 ± 9.2**^a^	**46.86 ± 4.1**^b^	**61 ± 9.6**^a,b^
**Serum AST (U/L)**	**145.25 ± 7.3**	**155.13 ± 14.05**^b^	**254.8 ± 17.9**^a^	**161.25 ± 21.3**^b^	**172.5 ± 16.7**^b^
**Serum creatinine (mg/dl)**	**0.26 ± 0.02**	**0.28 ± 0.05**^b^	**0.41 ± 0.04**^a^	**0.35 ± 0.05**^a,b^	**0.32 ± 0.04**^a,b^
**Serum urea (mg/dl)**	**29 ± 5.61**	**33.38 ± 3.3**^b^	**45.38 ± 4.14**^a^	**37.75 ± 4.77**^a,b^	**41.2 ± 3.5**^a^

^a^Significant difference compared to vehicle group

^b^Significant difference compared to positive control

#### Oxidative stress biomarkers

**Fig. 2:**
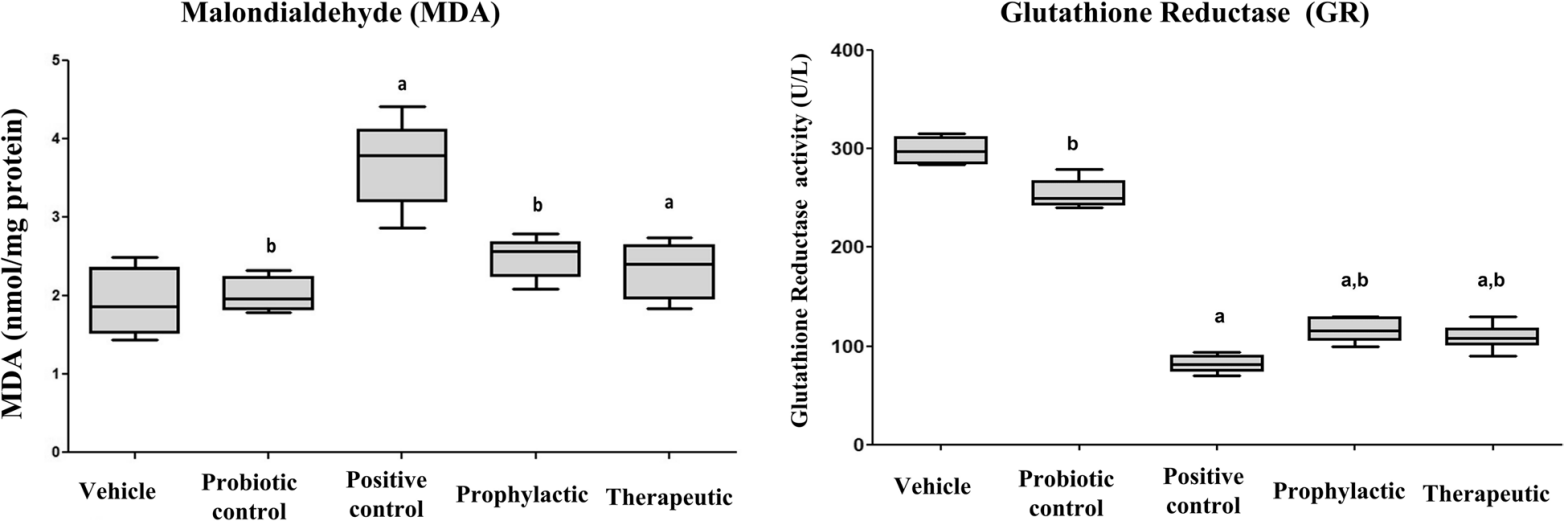
Effects of probiotic LGG administration on oxidant and antioxidant activity. Levels of malondialdehyde (MDA) and glutathione reductase (GR) were estimated in liver tissue homogenates of the different studied groups. Data presented as mean ± SD. ^a^Significant *p* < 0.05 compared to the vehicle group. ^b^Significant *p* < 0.05 compared to positive control

Cellular oxidative stress was evaluated in liver tissue homogenates, and the results revealed that the tissue level of the lipid peroxidation product, MDA, in the APAP-positive control group was significantly elevated (3.69 ± 0.5) compared with the vehicle group (1.9 ± 0.4). However, this elevation was found to be significantly reduced in the groups that received probiotic LGG either as a prophylactic (2.3 ± 0.2) or therapeutic (2.5 ± 0.2) compared with the APAP-positive control (3.69 ± 0.5) (Fig. 2). In contrast, measurement of the key cellular antioxidant marker GR in tissues revealed that APAP significantly (*p* < 0.05) reduced this antioxidant enzyme activity in the APAP-positive control group (81.2 ± 3.7 U/L) compared to the vehicle group (310 ± 20.3 U/L), respectively. However, administration of probiotic LGG was able to modulate this reduction to some degree as determined by the elevation of GR level in groups that received probiotic LGG either before or after APAP administration (Fig. 2), suggesting the ability of probiotic LGG to refine oxidative stress generated by APAP administration.

#### Inflammatory biomarkers

**Fig. 3:**
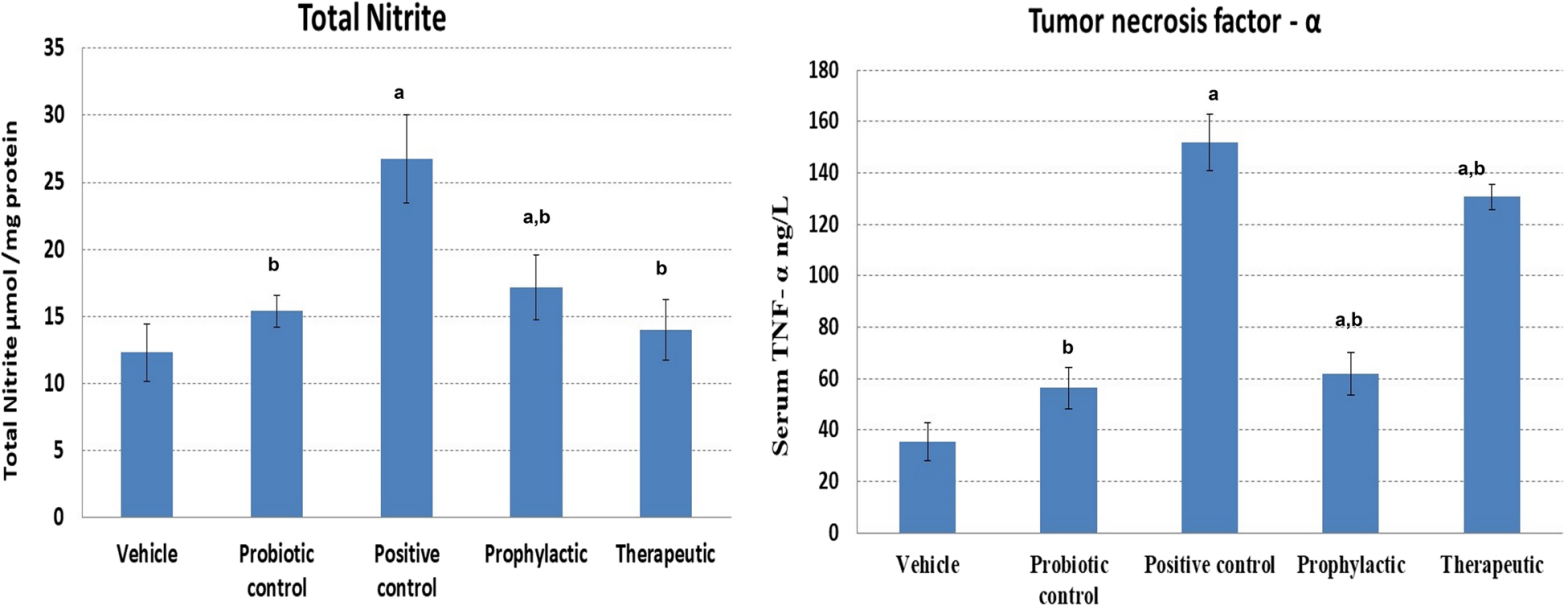
The modulatory effect of probiotic LGG administration on inflammatory markers. Total nitrite in liver homogenate (µmol/mg protein) and serum tumor necrosis factor-α (ng/ml) were measured in the different studied groups. Data presented as mean ± SD. ^a^Significant *p* < 0.05 compared to the vehicle group. ^b^Significant *p* < 0.05 compared to positive control

Enhanced inflammation associated with APAP hepatotoxicity was detected in our study (Fig. 3). The APAP-positive control group showed a significant increase in the levels of tissue nitrite and serum TNF-α (26.7 ± 5.2 µmol/mg and 151.8 ± 11 ng/L) respectively, compared with the vehicle group (12.3 + 2.1 µmol/mg and 35.3 ± 7.4 ng/L) respectively. This elevated state of inflammation was significantly decreased (*p* < 0.05) by the administration of probiotic LGG. In the prophylactic group, lower levels of tissue nitrite and TNF-α were observed (17.1 ± 2.4 µmol/mg protein and 61.88 ± 8.4 ng/L) respectively. This reduction in inflammatory biomarkers was also detected in the therapeutic group with total nitrite level of 14 ± 2.2 µmol/mg protein and TNF-α level of 130.7 ± 5 ng/L (Fig. 3).

### Enhancement effect of probiotic administration on the expression of Nrf2 and PGC-1α

**Fig. 4:**
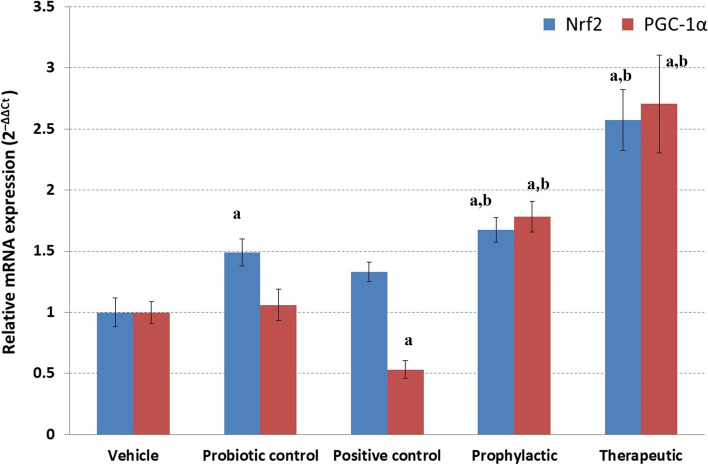
Probiotic LGG enhances the expression of Nrf2 and PGC-1*α*. Expression of Nrf2 and PGC-1*α* was detected in the liver of different studied groups using real-time PCR. Values were normalized to β-actin. Relative mRNA expression was calculated using the comparative 2^−ΔΔCt^ method. ^a^Significant *p* < 0.05 compared to the vehicle group. ^b^Significant *p* < 0.05 compared to positive control

The mRNA expression analysis of *Nrf2* revealed a significant elevation (*p* < 0.05) in its relative expression in all groups that received probiotic LGG compared with the vehicle group (Fig. 4). Moreover, APAP-treated rats that received probiotic LGG as a prophylactic or therapeutic showed a significant increase in *Nrf2* expression compared with the APAP-positive control group. This indicates the involvement of *Nrf2* in the prevention and treatment of APAP-induced hepatotoxicity.

The expression of *Nrf2* is under the control of the transcriptional coactivator *PGC-1a*. Therefore, we also estimated the expression of *PGC-1α*. Similar to the results for *Nrf2*, probiotic LGG was able to significantly enhance the expression of *PGC-1a* when administered to APAP-treated rats either as a prophylactic or therapeutic (Fig. 4).

### Effect of probiotic administration on the expression of protein kinase C (α and γ)

**Fig. 5:**
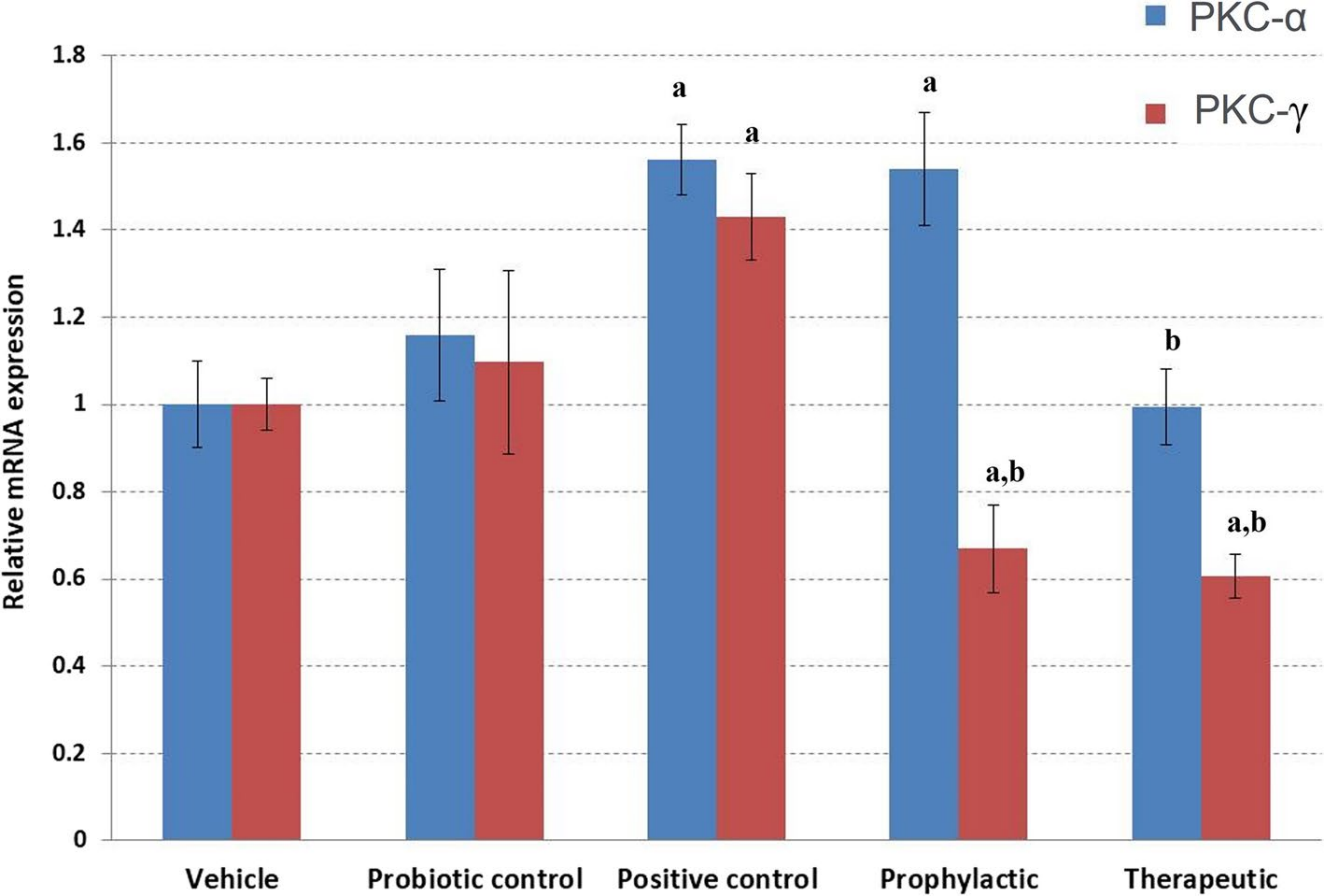
Relative expression of protein kinase C-alpha and -gamma in the liver of groups that received APAP and/or administered probiotic LGG. Expression values of PKC-alpha or gamma from RT-PCR were normalized to β-actin. Relative expression was calculated using the comparative 2^−ΔΔCt^ method. ^a^Significant *p* < 0.05 compared to the vehicle group. ^b^Significant *p* < 0.05 compared to positive control

APAP hepatotoxicity is mediated by a signal transduction pathway involving the activation of protein kinase C (PKC) family members. Therefore, we evaluated the differential expression of PKC family members alpha and gamma (α and γ) following probiotic LGG administration (Fig. 5). Our results revealed that the relative expression of both PKC (α and γ) significantly increased in the positive control group because of APAP-hepatotoxicity induction (Fig. 5). However, treatment with probiotic LGG was able to significantly (*p* < 0.05) reduce the elevated expression of PKC (α and γ) compared with APAP-positive control group. Regarding the prophylactic group, we detected a significant reduction in PKC-γ, but no significant decrease was detected for PKC-α. Based on our results, PKC-γ is the family member of PKC that is mostly affected by the probiotic LGG when administered as a prophylactic or therapeutic.

### Immunohistochemical analysis of Nrf2 protein expression in liver tissue sections

**Fig. 6:**
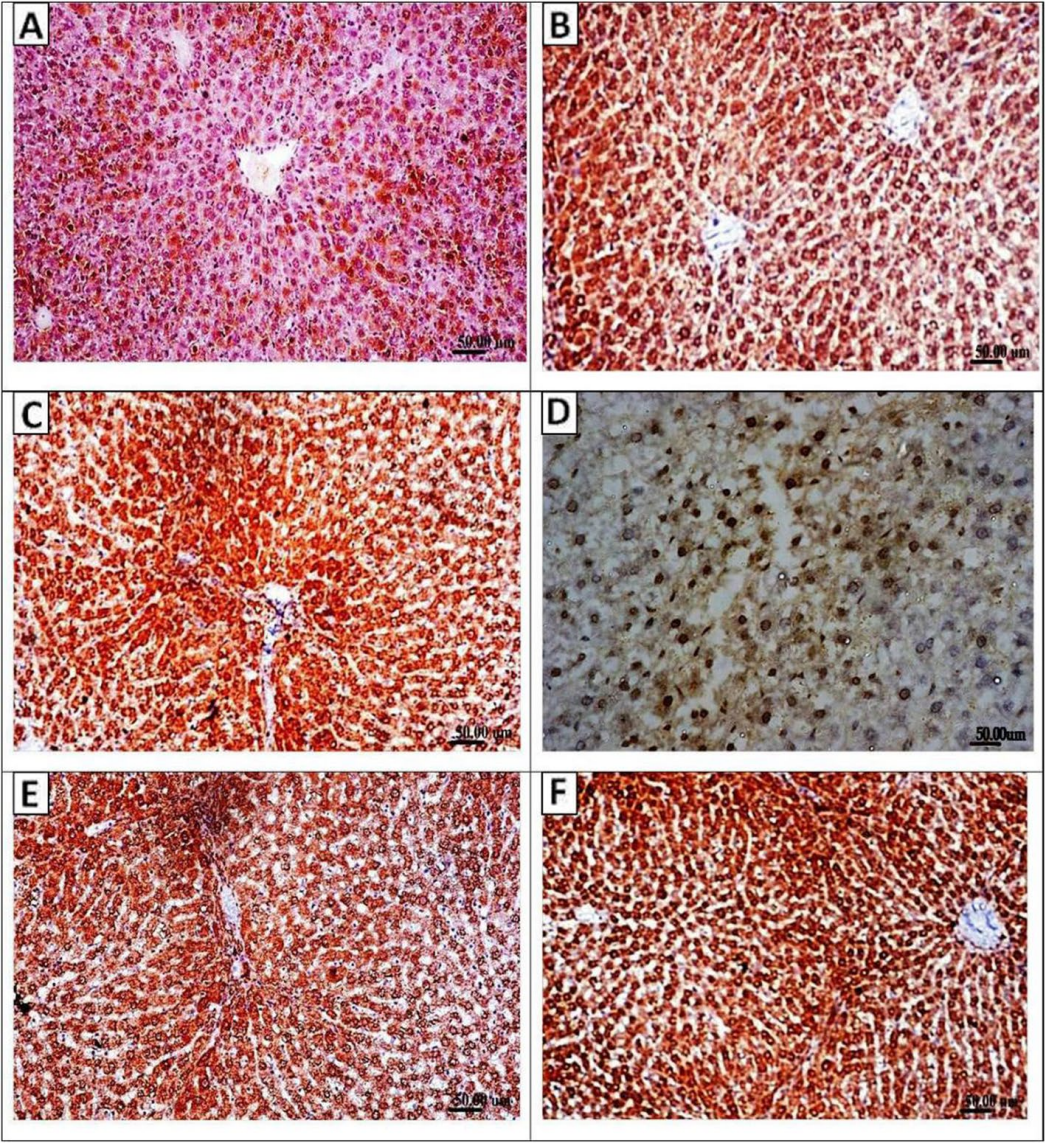
Representative images for immunohistochemical staining of Nrf2 expression in liver tissue sections of the different studied groups. Lower levels of Nrf2 protein expression were detected in the vehicle group (**A**). Mild to moderate Nrf2 expression scores in the probiotic control group (**B**) and positive APAP control group (**C**–**D**). Moderate to strong Nrf2 expressions were detected in rats that received probiotic LGG as a prophylactic (**E**) or as a treatment (**F**). (IHC × 200)

Examination of paraffin-embedded immune-stained liver sections for *Nrf2* protein expression showed results similar to that of real-time PCR. *Nrf2* protein was expressed in the hepatic cells of all groups but to a varying degree. While lower levels of *Nrf2* protein expression were observed in the vehicle group (Fig. 6A), liver sections from the probiotic control group showed moderate expression of *Nrf2* among the hepatic cells (Fig. 6B). Meanwhile, liver tissue sections from the positive control group exhibited increased expression of Nrf2 (Fig. 6 C and D) compared with that of the vehicle and probiotic control group. Administration of LGG probiotic either as a prophylactic or therapeutic following APAP injection significantly increased Nrf2 protein expression (Fig. 6 E and F), with intense staining observed for the therapeutic group (Fig. 6F).

## Discussion

The use of acetaminophen has dramatically increased, emerging as the most commonly utilized analgesic and antipyretic. Hence, APAP-related toxicity represents an intense healthcare problem, and it is ranked among the most common causes of toxicity worldwide [39]. Although the therapeutic window is narrow, the overdose of APAP is extremely hepatotoxic due to its ubiquitous nature and wide availability causing unintentional overdose [40]. In the present study, the pathological alterations in liver tissue sections of rats which received APAP were associated with severe congestion of the central veins, hepatic sinusoids, and a detected pericentral hepatocellular necrosis and peripheral vacuolar degeneration, indicating induced hepatotoxicity [41, 42]. Moreover, the significant elevation of liver enzymes ALT and AST indicates cellular leakage and loss of liver cell membrane functional integrity [43].

In the current study, the administration of probiotic LGG improved the histopathological features of liver tissue sections. Moreover, it significantly suppressed the elevation of serum liver enzymes, AST and ALT, toward normal levels in both groups that received LGG either as a prophylactic or therapeutic. This suggests that the hepatoprotective and therapeutic effect of probiotic LGG includes preserving the structural integrity of the hepatocellular membrane, thus preventing enzyme leakage into the blood circulation. These findings are considered healing signs of the hepatic parenchyma and hepatocyte regeneration [30, 44]. In our study, we noticed that the beneficial effect of LGG, in terms of histopathologic scoring, seems higher when used as a prophylactic agent, rather than as a treatment.

APAP-induced hepatotoxicity is initiated by the electrophilic metabolite NAPQI, which depletes glutathione in hepatocytes resulting in the elevation of mitochondrial oxidative stress. Consequently, reactive oxygen species (ROS) production is enhanced, and oxidative stress markers are elevated, accompanied by the depletion of antioxidants [41]. This is confirmed in the present study, in which APAP-induced hepatotoxicity was coupled with a significant elevation in lipid peroxidation and a simultaneous reduction in the activity of the antioxidant enzyme GR. The antioxidant property of probiotics, particularly LGG, and their powerful redox systems have been the focus of many studies over the past few years [45, 46]. The antioxidant ability of probiotic LGG is mainly related to its O_2_ tolerance and capacity to scavenge free radicals, chelate pro-oxidative metal ions, and enhance the production of antioxidant metabolites. Therefore, probiotics effectively support the redox balance in the human body. Moreover, in food manufacturing, the powerful redox system of probiotics can help extend the shelf life of food [47]. In the present study, we found that probiotic LGG, administered as therapeutic or prophylactic, modified all oxidative stress markers and increased the activity of antioxidant enzymes. Our findings are consistent with previous studies that reported the efficiency of probiotics, in general, in enhancing the total antioxidant capacity [48, 49].

Inflammation is considered a protective reaction against pathogens or chemicals to maintain body health. During inflammation, many cytokines are secreted and accumulated in the liver; among them, TNF-α is implicated as a critical mediator of APAP-induced hepatotoxicity [50]. In the present study, we found that the TNF-α levels significantly increased in all APAP-treated groups, an indication of a hepatic inflammatory response. In contrast, the administration of probiotic LGG was able to halt and manage the detected inflammatory state. Recent evidence highlighted the inhibitory effect of probiotics on TNF-α signaling through suppressing the phosphorylation of mitogen-activated protein kinase (MAPKs), c-Jun N-terminal kinases (JNK), and ERK signaling pathways [50]. In our study, we found that probiotic LGG, as a therapeutic or prophylactic agent, reduced the TNF-α levels in the APAP-induced liver injury model. In agreement with our finding, previous experimental models also support the anti-inflammatory efficacy of probiotics and the significant reduction in proinflammatory cytokines, including TNF-α, following probiotics administration [9, 51]. Interestingly, according to our data, the beneficial effect of LGG, in terms of TNF-α reduction, seems higher when used as a prophylactic agent, rather than as a treatment.

Nitric oxide is a well-established proxy of increased oxidative and inflammatory status. NO is a vital contributor to oxidative stress, and its level is positively correlated with ROS levels [52, 53]. On the other hand, APAP overdose can lead to hepatic necrosis, secondarily to oxidative stress, and enhance the release of cytokines by Kupffer cells [54]. According to our results, administration of LGG was able to significantly suppress the elevated levels of NO induced by APAP.

The transcriptional factor nuclear factor erythroid 2-like 2 (Nrf2) is the major regulator of the primary means of cellular defense and protects mammalian cells from chemical and oxidative damage. This has been highlighted in several investigations that have shown that Nrf2 knockout animals are more susceptible to xenobiotic-induced toxicity [55]. In the absence of stress, the activity and level of Nrf2 are suppressed by KEAP1 (Kelch-like ECH-associated protein 1), a cysteine-rich protein that functions as a substrate adaptor for CUL3-dependent ubiquitination of Nrf2, moving Nrf2 to proteasomal degradation. However, a chemical/oxidative stress can disrupt Nrf2 repression, allowing it to accumulate in the nucleus and transactivate the expression of cytoprotective genes and provoking an anti-inflammatory expression profile [55–57]. As an oxidative stress-mediated reaction, we found that the administration of probiotic LGG as a prophylactic or therapeutic was significantly associated with increased Nrf2 expression in the liver. Moreover, the combined administration of APAP with probiotic LGG as prophylaxis resulted in a higher significantly elevated expression of Nrf2 compared to its therapeutic effect.

Our findings can be interpreted in light of the results of previous studies highlighting the significant connection between the defense role of Nrf2 and probiotic administration [58]. Our findings are also consistent with previous studies linking probiotic supplementation to increased antioxidant activities of Nrf2 in different models and diseases [59–61].

Peroxisome proliferator-activated receptor-gamma coactivator 1 alpha (PGC-1α) is a key regulator of mitochondrial biogenesis and proliferation. PGC-1*α* has the ability to regulate the antioxidant activity of Nrf2 by suppressing the activity of glycogen synthase kinase 3β (GSK-3β). GSK-3β functions as an inhibitor of Nrf2 through phosphorylating Nrf2, thus suppressing its translocation to the nucleus. At the same time, Nrf2 regulates the transcriptional activity of PGC-1*α*. Accordingly, PGC-1*α* and Nrf2 synergistically participate in the regulation of antioxidant activity [62]. Moreover, the Nrf2/PGC-1*α* pathway regulates mitochondrial function and homeostasis. PGC-1α has a beneficial role against APAP-induced hepatotoxicity through the upregulation of Nrf2 and several antioxidant genes [63]. Therefore, the expression of both Nrf2 and PGC-1α works for supporting hepatoprotection. This explains the expression pattern detected for Nrf2 and PGC-1*α* in the present study, in which probiotic LGG was able to increase their expression, indicating an enhancement of antioxidant activity.

One of the interesting findings in our study is the contrast observed between Nrf2 and PGC-1α expression detected in the APAP-positive control group, where we observed a slight non-significant increase of Nrf2 directly following APAP-induced hepatotoxicity, and this could be explained as a first-line cellular adaptive response against the hepatotoxic drug APAP. On the other hand, we detected a suppression in the level of PGC-1α directly following APAP administration. Although it is not clear the reason behind this contrast, it is well established that hepatotoxicants can induce different responses of the cytoprotective modulator. In a previous study by Yan et al. [64], the expression level of PGC-1α decreased when detected at 6 h after APAP overdose, suggesting that mitochondrial biogenesis is disturbed by APAP at this time point. On the other hand, the study by Goldring et al. [65] reported that APAP activates Nrf2 to some extent in mouse liver following administration of non-hepatotoxic and hepatotoxic doses, implying that Nrf2 has a sudden response effect against APAP administration.

In addition to the oxidative stress pathway, studies suggest that upregulation of protein kinase C (PKC) mediates APAP-induced hepatotoxicity. PKCs are serine/threonine kinases that participate in several cellular signal transduction cascades. The PKC-α and γ isoforms are commonly expressed in liver tissue, and they are usually activated and involved in hepatotoxicity [66, 67]. Moreover, according to the study by Meng et. [68], PKC inhibitor treatment was protective against liver injury in diabetic rats. Based on the gene expression analysis in our study, the therapeutic administration of probiotic LGG resulted in notably lower levels of PKC-α and -γ in rats with APAP-induced hepatotoxicity.

## Conclusion

 The results of our study supports that probiotics have a therapeutic and protective role against APAP-induced hepatotoxicity. We found that pre-and posttreatment with probiotic *Lactobacillus rhamnosus* GG significantly enhanced antioxidant activities, reduced the level of oxidants, proinflammatory cytokines, and liver enzymes, and limited the pathological changes within the liver tissue. Moreover, our results revealed that pretreatment with *Lactobacillus rhamnosus* GG had more beneficial effects on the liver than a posttreatment regimen. Further studies are warranted to assess the other molecular pathways involved in the potential protective and therapeutic effect of probiotics against APAP-induced hepatotoxicity.

## Data Availability

All data generated or analyzed during this study are available from the authors upon reasonable request and with permission of the National Research Centre.

## Abbreviations

ALT: Alanine aminotransferase
APAP: N-Acetyl-p-aminophenol “acetaminophen”
AST: Aspartate aminotransferase
CO2: Carbon dioxide
GR: Glutathione reductase
H&E: Hematoxylin–eosin staining
JNK: C-Jun N-terminal kinases
LGG: *Lactobacillus rhamnosus* GG
MAPKs: Mitogen-activated protein kinase
MDA: Malondialdehyde
NAPQI: N-Acetyl-p-benzoquinone-imine
NO: Nitric oxide
Nrf2: Nuclear factor erythroid 2-like 2
PGC-1α: Peroxisome proliferator-activated receptor-gamma coactivator 1 alpha
PRKC-G: Protein kinase C-gamma
PRKC-α: Protein kinase C-alpha
ROS: Reactive oxygen species
TNF-α: Tumor necrosis factor-α
